# Effects of Ethanol Concentrations on Primary Structural and Bioactive Characteristics of *Dendrobium officinale* Polysaccharides

**DOI:** 10.3390/nu16060897

**Published:** 2024-03-20

**Authors:** Juan Yu, Yan Long, Jinyue Chi, Keyao Dai, Xiaoyu Jia, Haiyu Ji

**Affiliations:** 1 College of Life Sciences, Yantai University, Yantai 264005, China; yujuan14615@163.com (J.Y.); 18282780162@163.com (Y.L.); chijinyue@163.com (J.C.); 2 College of Food Science and Engineering, Tianjin University of Science and Technology, Tianjin 300457, China; dai13389086120@163.com; 3 Xinjiang Yuanxiang Agricultural Technology Co., Ltd., Hetian 848000, China; jiaxiaoyu331@outlook.com

**Keywords:** *D. officinale* polysaccharides, ethanol fractional precipitation, antitumor and antioxidant

## Abstract

Ethanol fractional precipitation can initially separate polysaccharides according to the structure, which exhibits strong correlation with the biological activities. This study aimed to investigate the impact of varying ethanol concentrations on the structural characteristics, and the antitumor and antioxidant activities of polysaccharides derived from *Dendrobium officinale* through ethanol fractional precipitation, as well as their internal relationships. The polysaccharides acquired by absolute alcohol additions at a final liquor-ethanol volume ratio of 1:1, 1:2, and 1:4 were named DOP-1, DOP-2, and DOP-4, and the supernatant was named DOP-S. The results of the structural analysis revealed that the increase in ethanol concentrations resulted in a reduction in the molecular weights and the acetylation degree of the polysaccharides, as well as a decrease in mannose content and an increase in glucose content. In vitro experiments demonstrated that DOP-S exhibited optimal antitumor and antioxidant activities. Animal experiments further confirmed that DOP-S suppressed the growth of solid tumors significantly, enhanced lymphocytes, mediated immune ability, and improved the activity of antioxidant enzymes. These findings would establish a theoretical foundation and provide technical support for further advances and applications of polysaccharides derived from *D. officinale* in the fields of food and medicine.

## 1. Introduction

*Dendrobium officinale* is a highly valued orchid with a homology for medicine and food. It is mainly composed of polysaccharides, flavonoids, and alkaloids, etc., and is valued commercially and medicinally in many Asian countries due to its therapeutic benefits [1,2]. The total flavonoids in *D. officinale*, counting for almost 4% [3], contributes to its strong antioxidant capacities [4]. The alkaloids belong to the terpenoid indole alkaloid class with contents of less than 1%, exhibiting certain efficacy in the control of tumors and cardiovascular diseases [5]. As reported, *D. officinale* polysaccharides accounted for more than 20% and primarily consist of 1,4-β-D-Man*p* and 1,4-β-D-Glc*p*, with minor amounts of arabinose, xylose, and galactose [6,7], as well as some acetyl groups [8], and exhibit multiple bioactivities including antitumor, antioxidant, immunomodulatory, anti-inflammatory, and so on [9,10,11]. However, the specific structure–activity relationship requires further investigation.

The chemical structure of polysaccharides is generally defined by their monosaccharide composition, configuration, position of glycosidic linkages, and other associated features. The structural properties of polysaccharides play a significant role in modulating their biological functions, which can be readily impacted under various extraction conditions [12]. The water extraction and alcohol precipitation method is an effective approach for the extraction and purification of target polysaccharides [13], utilizing the specific hydrogen bonding interactions between hydroxyl groups (-OH) in ethanol and in polysaccharide molecules, which would facilitate the aggregation and precipitation of polysaccharides [14]. The molecular weights of polysaccharides typically vary widely, and ethanol precipitation is widely recognized as an effective method for separating polysaccharides with diverse molecular weights [15]. Therefore, the polysaccharides of *D. officinale* were isolated through fractional precipitations in this study, followed by the analysis of the structural characteristics of obtained fractions.

Oxidative damage is intricately linked to the occurrence and progression of tumors [16]. Free radicals presented in intracellular and extracellular environments can promote the development of cancer cells via the introduction of genetic mutations, and regulate tumor cell proliferation and apoptosis by influencing the various signaling pathways [17,18]. Therefore, the simultaneous analysis of antioxidant and anti-tumor activities in vitro is commonly conducted by scholars when investigating the biological activity of natural substances [19]. However, often the impact of direct exposure to free radicals and tumor cells does not accurately reflect the practical applications of bioactive compounds from food raw materials, which indicates the necessity of conducting animal trials in order to ascertain the physiological effects within intricate biological milieu [20].

In the research presented, the method of ethanol fractional precipitation was employed to isolate four fractions of *D. officinale* polysaccharides, followed by the identification of the primary structure. In vitro/vivo antitumor and antioxidant experiments were conducted to comprehensively analyze the correlation among ethanol concentrations, polysaccharides structures, and biological activities. These findings would provide valuable data support for future research, and serve as a technical foundation for the further development and utilization of *D. officinale* in food and pharmaceutical industries.

## 2. Materials and Methods

### 2.1. Material and Chemicals

The dried stems of *D. officinale* were bought from Yunnan Shuairun Technology Co., Ltd. (Kunming, China), and smashed into a powder for further research. T-series dextrans were obtained from Sigma-Aldrich Co. (St. Louis, MO, USA). Monosaccharide standards, dimethyl sulfoxide (DMSO), and 3-(4,5-dimethyl-2-thiazolyl)-2,5-diphenyl-2-H-tetrazolium bromide (MTT) were purchased by Solarbio Biological Technology Co., Ltd. (Beijing, China). Antioxidant capacity assay kit (DPPH, ABTS method), glutathione peroxidase (GSH-PX) assay kit, superoxide dismutase (SOD) assay kit, and malondialdehyde (MDA) assay kit were purchased from Nanjing Jiancheng Bioengineering Institute (Nanjing, China). All the chemicals that remained were of analytical grade.

### 2.2. Isolation of Polysaccharides

**Figure 1:**
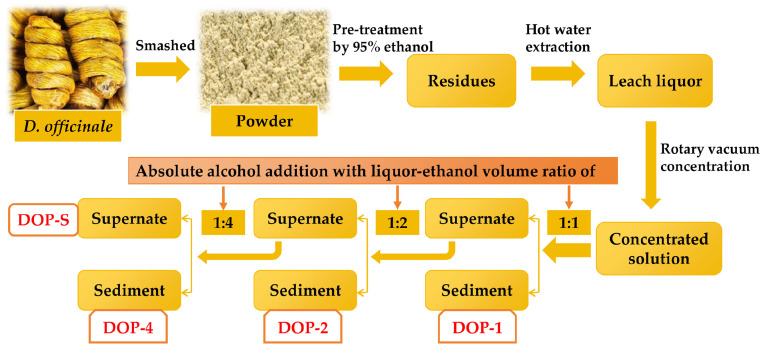
Preparation process of *D. officinale* polysaccharides.

The polysaccharides were fractionated from *D. officinale* by hot-water extraction and the ethanol precipitation method, which is presented in Figure 1. Briefly, the powder of *D. officinale* stems was subjected to three rounds of pre-treatment with 95% ethanol at room temperature for 12 h in order to eliminate pigments, lipids, and other small molecule substances. The residues were obtained by filtration and air drying, followed by hot water, then the leach liquor was collected and concentrated using a rotary vacuum evaporator at 60 °C. Subsequently, different fractions of polysaccharides were precipitated sequentially by adding absolute alcohol at final liquor-ethanol volume ratios of 1:1, 1:2, 1:4, named DOP-1, DOP-2, and DOP-4. Additionally, the supernatant after ethanol precipitation yielded another fraction of polysaccharides, named DOP-S. These four dried polysaccharide fractions were retained after lyophilization for further investigation.

### 2.3. Chemical Compositions Determination

The levels of total sugar, reducing sugar, and protein in the DOPs were determined using the phonol-sulphuric acid method; 3,5-dinitrosalicylic acid assay; and the C Brilliant Blue method, respectively [21]. Additionally, a Synergy HTX microplate reader (Bio-Tek, Charlotte, VT, USA) was utilized to obtain UV scanning spectra ranging from 200 nm to 600 nm.

The monosaccharide compositions of polysaccharide samples were analyzed using gas chromatography (GC) with slight modifications to the previously established method. In summary, 5.0 mg DOP samples were hydrolyzed with 2.0 mL of trifluoroacetic acid (TFA, 2 M) at a temperature of 110 °C for 3 h under nitrogen protection. Then they were acetylated at 90 °C for 30 min through the introduction of 10 mg hydroxylamine hydrochloride and 0.5 mL pyridine additions, and then acetic anhydride for an additional 30 min. The acetylated samples and standards were loaded to GC equipment with the HP-5 column following the detailed procedure [22].

### 2.4. The Molecular Weight Detection Assay

The average molecular weights of the DOPs were determined using high-performance liquid chromatography (HPGPC) with gel permeation chromatography column TSK-gel G4000PWxL and a refractive index detector, and the T-series dextrans with molecular weights were utilized as standards. Finally the molecular weights were calculated by establishing a standard curve and regression equation, plotting retention times on the X-axis and the logarithm of the molecular weights of T-series dextrans on the Y-axis [23].

### 2.5. Fourier Transformation Infrared (FTIR) Spectroscopy

The functional groups presented in the DOP samples were analyzed using the KBr pellet method with an FTIR spectrometer from Bruker, Germany. The key parameters used for the data acquisition and analysis included a 4 cm^−1^ resolution and 32 scan repetitions during a scan range of 4000–400 cm^−1^ [24].

### 2.6. Antioxidant Activity Assay In Vitro

The scavenging abilities of DOPs on DPPH and ABTS free radicals were assessed following the provided guidelines. Five different concentrations of DOPs (0.25, 0.5, 1, 1.5 and 2 mg/mL) were selected for the antioxidant investigation and the equivalent amounts of ascorbic acid were utilized as a positive control [22].

### 2.7. Antitumor Activity Assay In Vitro

The MTT assay was employed to determine the inhibitory effects of DOPs on H22 cells following previously established protocols. In brief, 1 × 10^4^ H22 cells were inoculated into each well of the 96-well plates, and incubated with different concentrations of DOPs for 24 h, followed by MTT (5 mg/mL) and DMSO additions, and the inhibitory rates of DOPs on H22 cells were evaluated at 490 nm [25].

### 2.8. Animal Experimental Design

Fifty female Kunming mice (8 weeks old, weighing 20 ± 2 g) were obtained from SPF Biotechnology Co., Ltd. in Beijing, China. The mice were housed in a controlled environment with relative humidity ranging from 45% to 55% and the temperature maintained between 20 and 25 °C. After an acclimatization period, the mice were randomly divided into the following five groups with 10 mice in each: blank group, model group, CTX group (treated with cyclophosphamide), low-dose DOP-S group (50 mg/kg of DOP-S), and high-dose DOP-S group (100 mg/kg of DOP-S). All animal experiments were carried out following the guidelines for animal research set by the scientific research management department of Tianjin University of Science and Technology.

**Figure 2:**
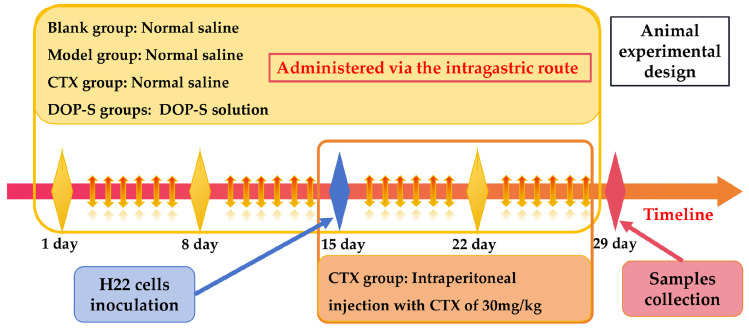
Schematic diagram of animal experimental design.

As shown in Figure 2, at the beginning, the blank and model groups were given 0.2 mL normal saline (0.9%) orally, whereas the low-dose and high-dose groups received DOPs at doses of 50 mg/kg and 1100 mg/kg respectively for 14 days. On day 15, all experimental groups except the blank group were injected subcutaneously into the right axilla with H22 cells of 2 × 10^6^ cells/mouse. The intragastric treatments continued for an additional 14 days in these groups, whereas the CTX group received daily intraperitoneal injections of CTX (30 mg/kg) for 14 days. At the end of the study, all mice were weighed and euthanized for further investigation.

### 2.9. Collection of Physiological Indicators

After the mice were euthanized, the thymus, spleen, and solid tumor were isolated, weighed, and recorded. The thymus and spleen indices (mg/g) were determined as the weights of thymus and spleen (mg) to the corresponding body weights (g). The tumor inhibitory rates (TIR, %) could be obtained with the subsequent formula:

TIR (%) = (W_0_ − W_1_)/W_0_ × 100. The variable W_0_ and W_1_ represented the mean tumor weight of the model group and the experimental group, respectively.

### 2.10. Leukocyte Subsets Analysis and Lymphocytes Proliferative Activity

The blood samples were promptly treated with EDTA-K_2_ to inhibit clotting, and subsequently subjected to analysis using an automated blood cell analyzer following protocols for mice, and the proportions of leukocyte subsets in each group of mice were determined and analyzed.

The splenic lymphocytes were aseptically prepared and inoculated into 96-well plates at a density of 1 × 10^6^ cells/well. Afterwards, individual additions of Concanavalin (Con A, 5 μg/mL) and lipopolysaccharide (LPS, 10 μg/mL) were conducted to each well in order to induce the proliferation of splenic T and B lymphocytes for 48 h, followed by MTT assay to evaluate the proliferative activities. The stimulation index (SI) was calculated as described below:

SI = OD_0_/OD_1_. The OD_0_ and OD_1_ were the absorbance (490 nm) of unstimulated wells, and Con A/LPS-stimulated wells, respectively.

### 2.11. Antioxidant Activity Determination In Vivo

The antioxidant enzymes activities and MDA levels in mice sera were assessed using the respective kits according to the provided Kit instructions.

### 2.12. Statistical Analysis

The data analysis was conducted using IBM SPSS 19.0 software. Statistical significance was assessed using the Student’s *t*-test, and the relevant results were reported as mean ± standard deviation. Differences were considered statistically significant when the *p* value was less than 0.05.

## 3. Results

### 3.1. Chemical Compositions of DOPs

**Figure 3:**
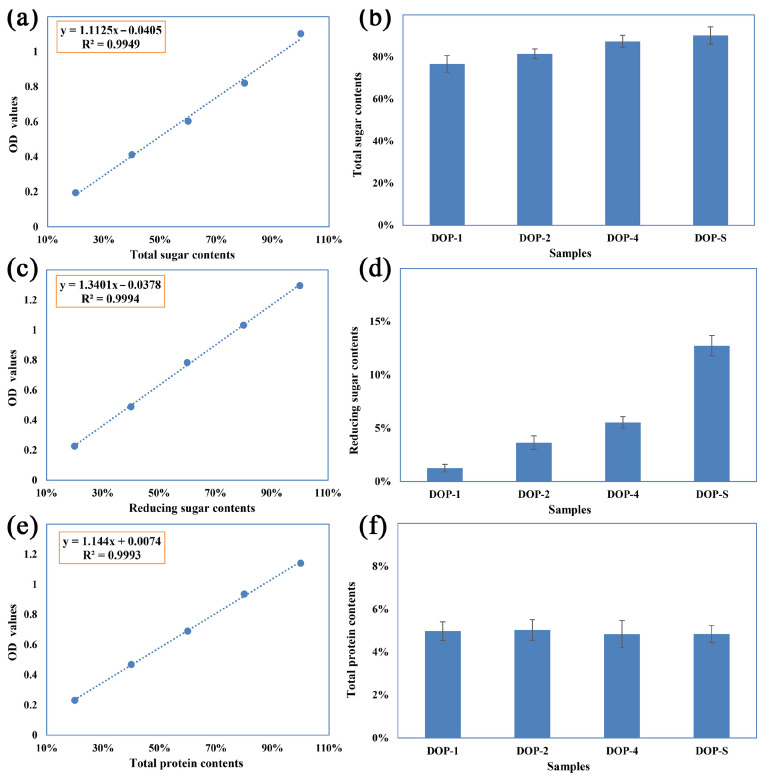
Chemical composition analysis of DOPs. Note: (**a**,**c**,**e**) demonstrates the standard curves of total sugar, reducing sugar, and protein contents; and (**b**,**d**,**f**) presents total sugar, reducing sugar, and protein in DOPs contents.

The total sugar, reducing sugar, and protein contents in DOPs were determined with the results presented in Figure 3. As displayed in Figure 3a,c,e, standard curves of total sugar, reducing sugar, and protein contents were calculated as follows: y = 1.1125x − 0.0405, R^2^ = 0.9949; y = 1.3401x − 0.0378, R^2^ = 0.9994; y = 1.144x + 0.0074, R^2^ = 0.9993, wherein the x and y represent the chemical compositions and the corresponding OD values. The high R^2^ values indicated favorable linearity in the standard curves and high accuracy in the measurement results. The relevant chemical compositions of the DOPs are presented in Figure 3b,d,f. With the increase in ethanol concentrations, the obtained DOP-1, DOP-2, DOP-4, and DOP-S showed progressively increased water solubility. Consequently, there was a corresponding increase in the total sugar contents of these fractions, which might be attributed to the variations in the complexity of monosaccharide compositions and molecular weight distributions. The reducing sugar contents presented a more obvious increasing trend as the ethanol concentrations increased, indicating the lower molecular weight distributions. However, the protein contents of these components were significantly low, constituting approximately 5%, and there was no discernible variation observed among different samples, which could potentially be attributed to the denaturation of proteins induced by the pretreatment with 95% ethanol or an inherently low protein content within *D. officinale*.

### 3.2. Ultraviolet Full Wavelength Spectra of DOPs

**Figure 4:**
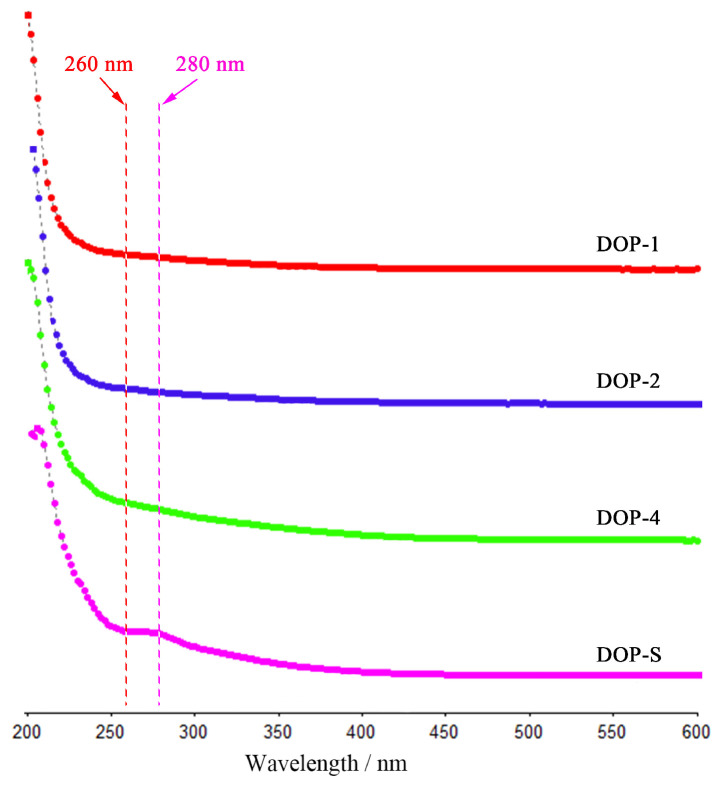
Ultraviolet full wavelength spectra of DOPs scanning from 200 to 600 nm.

Figure 4 displays the ultraviolet full wavelength spectra of DOPs, which could further estimate the purity via the absorption intensity of nucleic acids (260 nm) and proteins (280 nm) [26]. As shown, the absence of any discernible impurity absorption peaks in these four fractions suggests relatively high polysaccharide contents, which aligns with the findings of the chemical composition analysis. In addition, the weak absorption peak at around 210 nm might be caused by the higher contents of total polysaccharides and reducing sugars in DOP-S [27]. Finally, these spectra indicate that the DOPs showed high purity, which was convenient for the following structure and activity analysis.

### 3.3. Molecular Weight Distributions of DOPs

**Figure 5:**
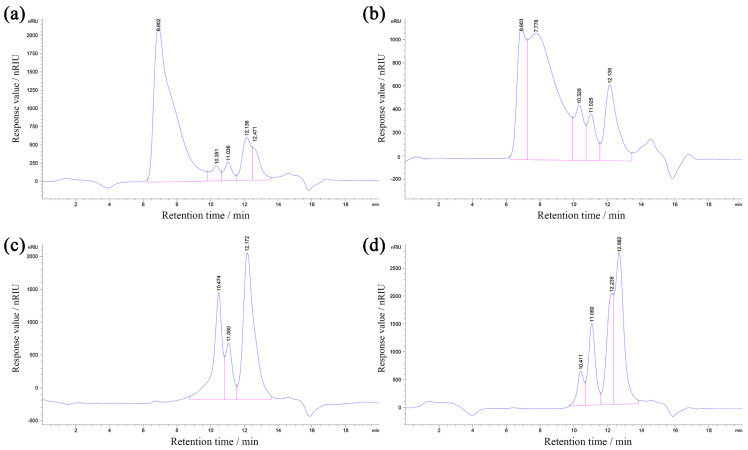
HPGPC files of DOP-1 (**a**), DOP-2 (**b**), DOP-4 (**c**), and DOP-S (**d**).

The HPGPC files of the DOPs, which could reflect the molecular weight distributions, are displayed in Figure 5. The molecular weights of the DOPs would be decreased with the prolonging of retention time, wherein the peak areas positively correlated with the polysaccharide contents of corresponding molecular weights. As presented, the elongation of the retention time of separated polysaccharide components was observed as a consequence of the higher ethanol concentration, suggesting the decrease in the distributions of molecular weights, which was consistent with previous results [28].

### 3.4. Monosaccharide Compositions of DOPs

**Figure 6:**
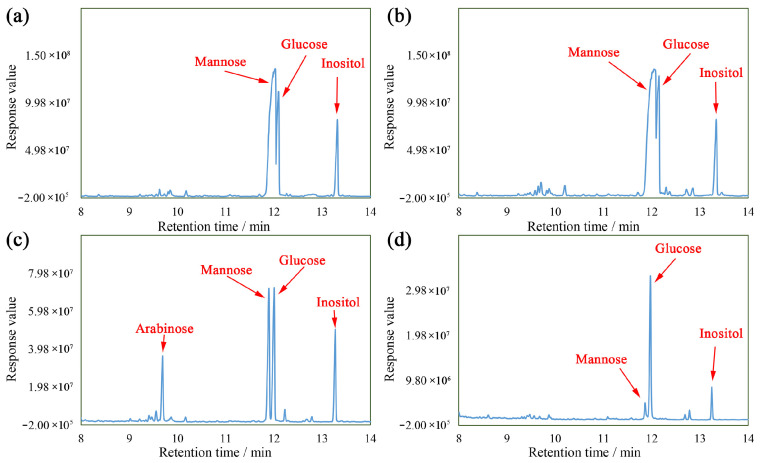
Monosaccharide compositions of DOP-1 (**a**), DOP-2 (**b**), DOP-4 (**c**), and DOP-S (**d**).

Figure 6 displays the monosaccharide compositions of DOPs. As exhibited, these fractions primarily consisted of mannose and glucose, except for some arabinose in the DOP-4. The DOP-1, DOP-2, DOP-4, and DOP-S were comprised of Man:Glc (molar ratio of 3.38:1), Man:Glc (molar ratio of 3.18:1), Man:Glc:Ara (molar ratio of 1.02:1:00:0.43), and Man:Glc (molar ratio of 0.12:1), respectively, indicating that the solubility and molecular weights of the DOPs exhibited an inverse correlation with the mannose contents, whilst demonstrating a positive correlation with the glucose contents.

### 3.5. Functional Groups of DOPs

**Figure 7:**
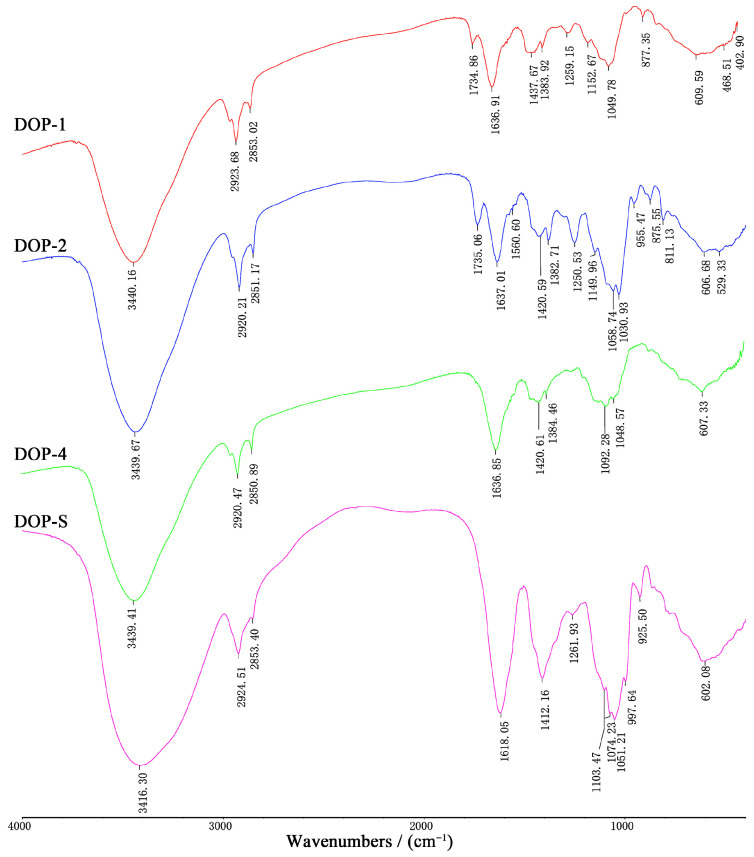
FTIR spectra of DOPs.

The characteristic functional groups of DOPs were analyzed via FTIR and the results are presented in Figure 7. As shown, these four samples all exhibited obvious absorption peaks at 3400~3450 cm^−1^ and 2850~2950 cm^−1^, which corresponded to the stretching vibrations of O–H bonds and C–H bonds respectively, suggesting the characteristic absorption peaks of polysaccharides [29,30]. In particular, the peaks observed at around 1735 cm^−1^ in DOP-1 and DOP-2 could be attributed to the vibrational motion of the esterified carboxyl groups’ carbonyl double bonds (C=O) in an asymmetric stretching manner [31], indicating the acetyl modification located at O–2 of Man*p* and Glc*p* [32]. The similar absorption peaks detected at around 1640 cm^−1^ and 1420 cm^−1^ were indicative of the vibration associated with the O–H bonds of bound water and C–O linkages [33,34]. It was worth emphasizing that absorption peaks representing O–H functional groups (3416 cm^−1^ and 1618 cm^−1^) in DOP-S exhibited significant signals enhancement and blue shift, indicating an increased affinity for water [35]. The spectral region between 1200 cm^−1^ and 1000 cm^−1^ was associated with vibrations related to C–O–C glycosidic bonds and asymmetric stretching vibrations of C–O–H side groups [36], as well as the existence of pyranose rings [37], thus the relevant weak absorption peaks in DOP-4 could be ascribed from the furanose configuration of arabinose. The presence of β- and α-glycosidic bonds was indicated by the absorption peaks observed around 930 cm^−1^ and 870 cm^−1^, respectively [38], suggesting improved β-glycosidic bond contents in these fractions with the increase of ethanol concentrations. Therefore, these results revealed distinct polysaccharide characteristics in DOP-1, DOP-2, DOP-4, and DOP-S. Whereas DOP-1 and DOP-2 presented acetylation modifications, potentially accounting for their limited solubility, DOP-4 demonstrated relatively low pyranose content, whereas DOP-S exhibited strong water affinity and β-glycosidic configuration characteristics.

### 3.6. Antitumor Activities of DOPs In Vitro

**Figure 8:**
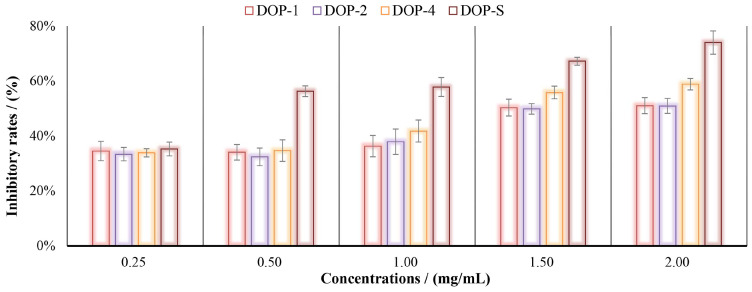
Inhibitory rates of DOPs on H22 cells in vitro.

The utilization of in vitro cell culture experiments is frequently employed for the efficient screening of antitumor drugs. Figure 8 demonstrates the inhibitory rates of DOPs on H22 cells at concentrations of 0.25, 0.50, 1.00, 1.50, and 2.00 mg/mL. As shown, these components exhibited certain inhibitory effects on H22 cells proliferation in vitro, dose-dependently. Among them, the alcohol-soluble component DOP-S presented the best tumor cell proliferation inhibition rate, reaching 74% at 2.00 μg/mL, which might be attributed to the excellent water solubility and strong permeability.

### 3.7. Antioxidant Activities of DOPs In Vitro

**Figure 9:**
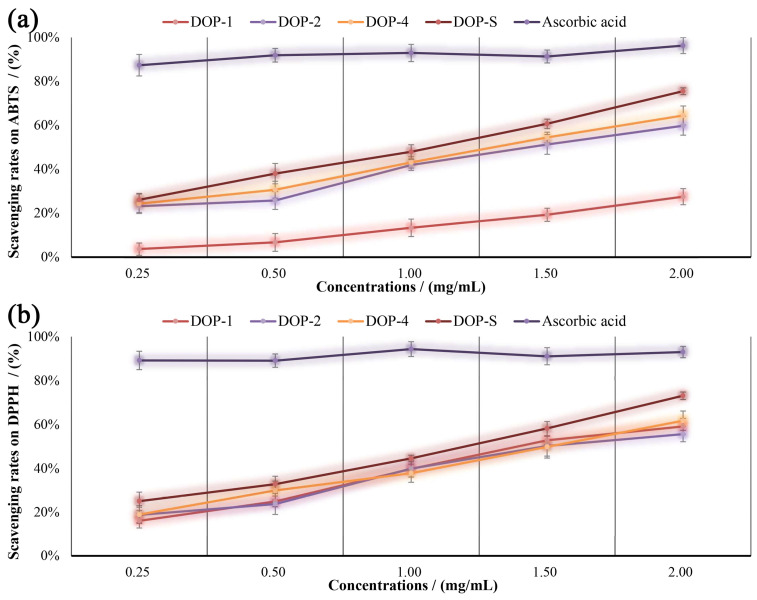
Scavenging rates of DOPs on ABTS (**a**) and DPPH (**b**) radicals in vitro.

The antioxidant activity of various chemical compounds is commonly evaluated using stable free radicals ABTS and DPPH [39]. Figure 9a,b demonstrates the scavenging effects of DOPs on the ABTS and DPPH at concentrations of 0.25, 0.50, 1.00, 1.50, and 2.00 mg/mL. As presented, the scavenging abilities of DOPs on these free radicals were significantly increased as the dosage increased, and the DOP-S fraction exhibited the superior in vitro antioxidant effect, achieving a maximum scavenging rate of 75.5% for ABTS and 73.1% for DPPH.

### 3.8. Physiological Indicators Collection of H22-Bearing Mice

**Figure 10:**
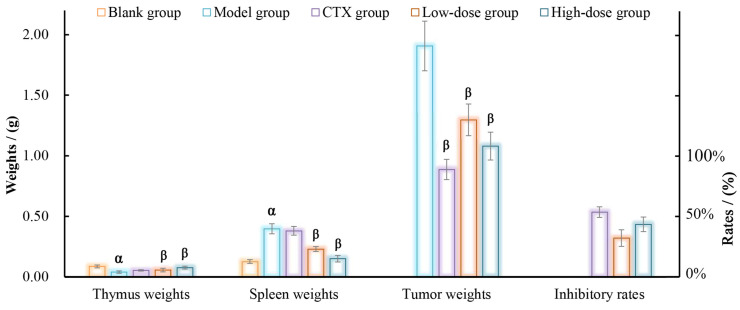
Effects of DOP-S on immune organs and H22 solid tumors. Note: ^α^ *p* < 0.05, vs. blank group; ^β^ *p* < 0.05, vs. model group.

The assessment of in vitro activity tests often proves to be highly effective and expeditious, thereby providing fundamental data for subsequent animal experimentation. In the present study, DOP-S demonstrated both superior in vitro antitumor and antioxidant effects compared with the other fractions, thus the tumor mice model was established for the DOP-S activities evaluation, and the effects of DOP-S on immune organs and H22 solid tumors are displayed in Figure 10.

The thymus and spleen possess impressive abilities to adapt, guaranteeing efficient performance throughout different life phases via regulating immune responses, and the weights could be used as partial indicators of the organism’s immune capacity [40]. As observed, the mice in the model group displayed notable impairment in the major immune organs due to the excessive growth of H22 tumor cells, leading to a significant decrease in thymus weights and a substantial increase in spleen weights compared with the blank group. Moreover, the average weight of solid tumors reached 1.91 g. However, upon administration of DOP-S, there was a remarkable improvement in immune organ weights compared with the model group, and the average tumor weights decreased to 1.30 g and 1.08 g, with tumor inhibitory rates of 32% and 43%, respectively. As expected, in the CTX group, the weights of the immune organs were not significantly improved, whereas the weights of the tumors exhibited a significant reduction compared with the model group, suggesting that CTX demonstrated potent toxicity towards both tumor cells (inhibitory rate of 54%) and immune organs.

### 3.9. Effects of DOPs on Lymphocytes Activities

**Figure 11:**
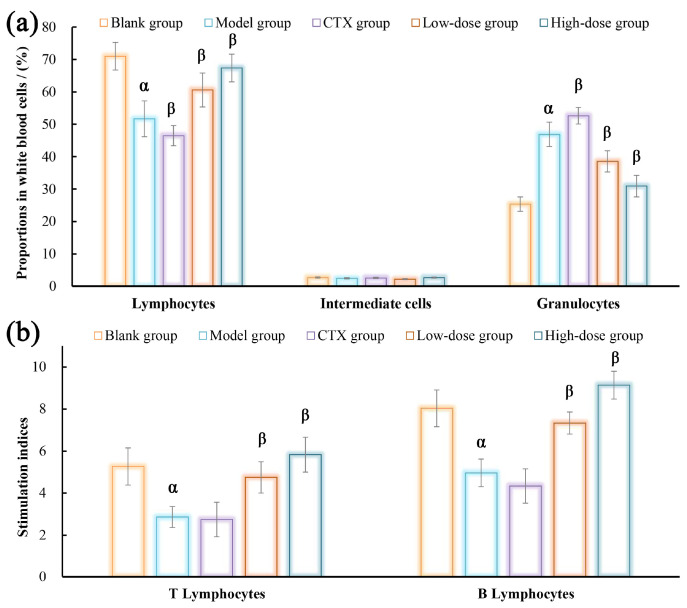
Effects of DOPs on lymphocyte proportions (**a**) and activities (**b**). Note: ^α^ *p* < 0.05, vs. blank group; ^β^ *p* < 0.05, vs. model group.

The lymphocytes proportions in the blood and the proliferative activities in the spleen were assessed for each group of mice, with the results depicted in Figure 11. As presented in Figure 11a, compared with the blank group, the model group demonstrated a remarkable reduction in the proportions of lymphocytes among peripheral blood leukocytes, accompanied by a notable increase in the proportions of granulocytes, suggesting malignant tumor proliferation induced inflammation and immunosuppression. The administration of DOP-S resulted in a significant improvement in the proportions of lymphocytes and a significant reduction in the proportion of granulocytes, indicating effective control of mice tumors compared with the model group.

Figure 11b demonstrates the proliferative capacities of T/B lymphocytes in the spleen, which were stimulated by Con A and LPS, respectively. As presented, both T and B cell activities obviously descended in the model group due to the tumor growth compared with the blank group, suggesting the inhibition of tumor-specific immune responses mediated by lymphocytes. Compared with the model group, the DOP-S group presented remarkably enhanced proliferative activities of lymphocytes, suggesting that DOP-S could enhance the tumor-induced immune injury to a certain extent. However, a mature chemotherapeutic agent, CTX, induced anticipated lymphocyte damage in terms of both quantity and viability within the tumor-bearing mice.

### 3.10. Antioxidant Activities of DOPs on H22-Bearing Mice

**Figure 12:**
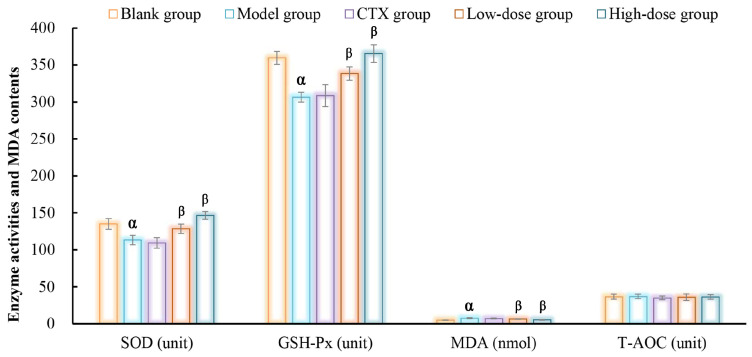
Antioxidant activities of DOPs on H22-bearing mice. Note: ^α^ *p* < 0.05, vs. blank group; ^β^ *p* < 0.05, vs. model group.

The uncontrolled proliferation of tumor cells can result in excessive generation and accumulation of reactive oxygen species, and results in oxidative damage to cellular components. In this study, the antioxidant capacities of DOP-S on blood in tumor-bearing mice were detected with the results presented in Figure 12. As displayed, the activities of SOD and GSH-Px in the model group were obviously inhibited compared with the blank group, whereas the contents of MDA were obviously increased, indicating the tumor growth induced oxidative injury. Compared with the model group, the DOP-S could significantly enhance these enzymes’ activities whilst suppressing the MDA levels in tumor-bearing mice. However, the total antioxidant capacities (T-AOC) exhibited no significant differences amongst these groups, which might be attributed to the powerful self-regulatory capacity of peripheral blood.

## 4. Discussion

Ethanol fractional precipitation is a commonly employed primary separation technique for polysaccharides, and precipitated fractions at different ethanol concentrations often exhibit distinct variations in structural characteristics including molecular weights distributions, monosaccharide compositions, and functional group contents [41]. The published literatures indicated that there was a notable decrease in the molecular weight distributions of polysaccharides from various materials as the ethanol concentrations increased, thereby exerting a significant impact on biological activities [42,43]. In the present study, the structural properties of four fraction samples from *D. officinale* were determined, and the results revealed that the distributions of molecular weights were obviously reduced, which also aligned with existing literature [44]. However, it is not possible to draw a definitive conclusion regarding the impacts of ethanol concentrations on the monosaccharide compositions and glycosidic bond configurations of polysaccharides due to the significant variations in composition amongst different raw materials. The contents of mannose and α-glycosidic bonds in the obtained fractions of *D. officinale* polysaccharides gradually decreased whereas the proportions of glucose and β-glycosidic bonds were improved with an increase of ethanol concentration.

The acetylation is the most prevalent modification for plant polysaccharides, which regulates the physicochemical characteristics and protects plant cell walls from microbial hydrolytic enzymes [45]. In addition, natural and chemical acetylation exhibits an ability to modify the conformation of polysaccharide chains, resulting in an increased exposure of polar groups, which holds promise for enhancing immunomodulatory, antioxidant, and other related activities [46]. Coincidentally, *D. officinale* polysaccharides are abundantly present in natural acetylation [7], and the isolation process is significantly influenced by the concentration of ethanol, resulting in the distinct acetyl absorption peaks in DOP-1 and DOP-2 at around 1730 cm^−1^ of FTIR spectra. These results could provide data and technical support for the preparation of natural acetylated polysaccharides in *D. officinale*.

The current scientific system extensively employs in vitro experiments for the evaluation of primary biological activities screening, owing to the exceptional efficiency and practical effectiveness [47,48]. Therefore, the direct interaction of active compounds against free radicals and tumor cells have become a widely employed approach for evaluating the primary antioxidant and antitumor activities [19,49]. In this study, with the increase of ethanol concentrations, the antioxidant and anti-tumor effects of the obtained fractions were gradually improved, suggesting that DOP-S exhibited the highest levels of antioxidant and antitumor bioactivity in vitro.

The in vitro experiments cannot be completely reflected in the complex in vivo environment, leading to deficiencies in the evaluation process of active substance efficacy, thus animal experiments are still necessary in order to further confirm the bioactivities of the bioactive components [50,51]. Therefore, the in vivo antioxidant and antitumor activities of DOP-S were evaluated via tumor animal model construction. The occurrence and progression of tumors, as well as the chemotherapy treatments, can induce immune dysfunction and oxidative stress, thereby exacerbating the overall health conditions of the body [52,53]. In addition, excessive accumulation of free radicals can equally damage both immune cells and tumor cells. Unfortunately, the high expressions of GSH in tumor cells leads to their increased resistance against oxidative damage [54]. Consequently, oxidative stress is more likely to impair the immune system and induce apoptosis in normal cells, thereby creating favorable conditions for tumor regeneration [55]. There is an inseparable intrinsic relationship between antioxidant and anti-tumor activities, leading to the necessity of the joint investigation and development [56]. In this study, the DOP-S could remarkably protect the thymus and spleen in tumor-bearing mice, and suppress the solid tumor growth with an inhibitory rate of 43%, and moreover, improve the proportions and proliferation capacities of T and B lymphocytes. In addition, DOP-S also significantly enhanced the bioactivities of antioxidant enzymes, resulting in low levels of MDA.

## 5. Conclusions

In conclusion, the four fractions derived from *Dendrobium officinale* were prepared via ethanol fractional precipitations, named DOP-1, DOP-2, DOP-4, and DOP-S. The results revealed that with the increase of ethanol concentrations, the obtained fractions presented reduced molecular weight distributions, mannose proportions, acetylation degrees, as well as increased water solubility and glucose contents. DOP-S displayed superior antitumor and antioxidant effects in vitro, which might be attributed to the structural characteristics. In addition, the animal experiment results demonstrated that DOP-S could significantly suppress tumor growth with an inhibitory rate of 43% via protecting immune organs, enhancing lymphocytes proportions and activities, and improving antioxidant enzymes activities. These data would facilitate the further development and application of polysaccharides from *D. officinale* in the food and medicine industries.

## Data Availability

The data presented in this study are available within this article.
